# Quantitative evaluation of binary digital region asymmetry with application to skin lesion detection

**DOI:** 10.1186/s12911-018-0641-7

**Published:** 2018-06-27

**Authors:** Agustin Sancen-Plaza, Raul Santiago-Montero, Humberto Sossa, Francisco J. Perez-Pinal, Juan J. Martinez-Nolasco, Jose A. Padilla-Medina

**Affiliations:** 1 Department of Engineering Sciences, Tecnologico Nacional de Mexico-Instituto Tecnologico de Celaya, Antonio Garcia Cubas 600, Fovissste, 38010 Celaya, Mexico; 2 Department of Computer Science, Tecnológico Nacional de México-Instituto Tecnológico de León, Av. Tecnológico S/N, Fracc. Industrial Julián de Obregón, 37290 León, Mexico; 3 Department of Computer Science, Instituto Politécnico Nacional-Centro de Investigación en Computación, Av. Juan de Dios Bátiz S/N, Nueva Industrial Vallejo, 07738 Ciudad de México, Mexico

**Keywords:** Melanoma asymmetry measurement, Shape analysis, Digital binary region, Image processing, Computer aided diagnosis system

## Abstract

**Background:**

The performance of Computer Aided Diagnosis Systems for early melanoma detection relies mainly on quantitative evaluation of the geometric features corresponding to skin lesions. In these systems, diagnosis is carried out by analyzing four geometric characteristics: asymmetry (A), border (B), color (C) and dimension (D). The main objective of this study is to establish an algorithm for the measurement of asymmetry in biological entities.

**Methods:**

Binary digital images corresponding to lesions are divided into 8 segments from their centroid. For each segment, the discrete compactness value is calculated using Normalized E-Factor (NEF). The asymmetry value is obtained from the sum of the square difference of each NEF value and corresponding value of its opposite by the vertex. Two public skin cancer databases were used. 1) Lee’s database with 40 digital regions evaluated by fourteen dermatologists. 2) The PH^2^ database which consists of 200 images in an 8-bit RGB format. This database provides a pre-classification of asymmetry carried out by experts, and it also indicates if the lesion is a melanoma.

**Results:**

The measure was applied using two skin lesion image databases. 1) In Lee’s database, Spearman test provided a value of 0.82 between diagnosis of dermatologists and asymmetry values. For the 12 binary images most likely to be melanoma, the correlation between the measurement and dermatologists was 0.98. 2) In the PH^2^ database a label is provided for each binary image where the type of asymmetry is indicated. Class 0–1 corresponds to symmetry and one axis of symmetry shapes, the completely asymmetrical were assigned to Class 2, the values of sensitivity and specificity were 59.62 and 85.8% respectively between the asymmetry measured by a group of dermatologists and the proposed algorithm.

**Conclusions:**

Simple image digital features such as compactness can be used to quantify the asymmetry of a skin lesion using its digital binary image representation. This measure is stable taking into account translations, rotations, scale changes and can be applied to non-convex regions, including areas with holes.

## Background

A Computer Aided Diagnosis System (CADS) requires a priori information to improve its analytical process and performance in the diagnosis of a variety of diseases. This leads to better decision making and improved patient care.

One of the main components of many CADS is the quantification of the asymmetrical shape of the biological entity under study. The measured degree of asymmetry is very important in several areas of medical biology, where the variation in the morphology of a biological entity can be related to the presence of a pathology. For example, Karnan and Thangavel [1] used geometric asymmetry to detect microcalcifications in breast cancer. Additionally, in the area of jaw correction, asymmetry is used to measure the progress of orthodontic treatment [2], while Ercan measured young people’s health using the asymmetry of their faces [3].

The quantitative evaluation of this feature is crucial in dermatology, in which the CAD system is used as the highest criterion for diagnosing a malignant lesion [4]. There are different approaches for measuring the asymmetry of biological entities, the most common being the approach that computes the asymmetry over a binary digital image. All approaches are based on first locating the digital region centroid, and then calculating the major axis of that region.

It is common that the shape appears rotated on the major axis angle, with the major axis fixed horizontally, the minor axis bisecting the major axis [5], and the digital region divided into four sections.

An algorithm was used to calculate the computational load required to transform an irregular polygon to a regular polygon, asymmetry to symmetry transformation was used as an asymmetry parameter. However, as the algorithm relies on image resolution, it is not invariant to scale transformations.

Several authors [6–8] define asymmetry measurement where asymmetry is evaluated by using the difference in area among *N* sub-regions. These are obtained from a digital region variance of *M* segments. The main drawback of these methods is their dependence of the resolution related to digital region.

In the Stoecker’s proposal [9] shape was shifted so that x and y coordinates of the image coincide with the centroid of image, then the shape is rotated to align with centroidal principal axes. Finally, the shape was divided in four sub-regions, rotation angle of digital region. The asymmetry value is given by subtracting shape area on one side of the axis from the reflected shape which results in two area differences,

1 $$ Asymmetry\_ Ref=\frac{\Delta  {A}_{min}}{A_{total}}\ast 100 $$

where *∆A*_*min*_ is the lowest absolute value difference between subregions and *A*_*total*_ is the area of shape. A similar method is presented in [10], in which major and minor axes are used to generate eight sub-regions to extract three shape descriptors: perimeter, area and classic compactness or thinness ratio.

Following the procedure described above, a set of 24 features is used to create a description vector of the digital region. Each feature is calculated by Eq. 2:

2 $$ {R}_i=\frac{Q_i}{\sum_{i\ne i}{Q}_j} $$

where *R*_*i*_ is ratio of features and *Q*_*i*_ is vector feature in quadrant *i*.

In [11] a color image rotation of a skin lesion on the major axis and grid is superimposed onto the color image, producing a new image; however, each new pixel or cell contains the mean value of the pixel found inside in the area of the cell grid. The distance between opposite cells on the border and the major axis is then calculated, while the sum of the difference between these distances is used as an asymmetry measurement.

In contrast, Santiago-Montero [12] avoids the pro-cess of finding the major axis and rotating the digital region, instead using the centroid position to segment the digital region into four sectors and then calculating a compactness value, which is expressed by using perimeter ratios.

The combinatorial sum of the quadratic differences of these four values is used to measure asymmetry. Other studies have focused on finding the best axes of asymmetry that could be used to obtain a better subdivision of the digital region [8, 13, 14].

For instance, Clawson et al. [8] performed a transformation of the digital region to frequency domain and applied the Fourier transform to calculate the major axis in the space domain. Cudek exhaustively searched and tested all possible axes at intervals of 1, 2 and 4 degrees [13, 14]. The digital region is classified into three possible categories according to the clinical criteria for regions with 1, 2 or 0 asymmetry axes.

Liu et al. [15] use a color image to produce a 3D intensity map, while the region asymmetry is deter-mined by the qualitative evaluation of the regularity of a 3D surface. Ma et al. [16] undertake a digital region transformation by using a function called Relative Radial Distance. In this representation, the axes are obtained to subdivide the region into four sectors, after which, the fractal relationship is used to calculate the differences between them.

It should be noted that, with the exception of [11, 15], color is the main variable used to calculate asymmetry. In general, the reported approaches work with the binary representation of the skin lesion contained in a digital region, obtained without the complete automatic process used in many approaches. In addition, several papers do not explain the process of generating binary image databases, such as Lee and PH^2^ database [17, 18], in that studied the binary representation of the skin lesion was usually hand made. This way to process the segmentation of skin lesion generates smoothing borders, and the regions are adjusted to a convex region. These drawbacks are the causes different measurements would not to work correctly.

On the other hand, several CAD systems use the Total Dermatoscopic Value (TDV) to calculate whether or not a skin lesion is a melanoma, in which the evaluation of the asymmetry of a binary digital region plays a major role. The most common expression of TVD is given by:

3 $$ TDV=1.3\ast A+0.1\ast B+0.5\ast C+0.5\ast D $$

where the asymmetry, *A*, is more important than the border (*B*), color (*C*) and dimension(*D*) [19].

This study presents a digital topology-based approach which is used to obtain a quantitative value for the asymmetry of the binary digital regions. This method confirms that a robust description of the asymmetry can be generated using only the eight subdivisions generated by the eight adjacencies. A simple shape descriptor for region compactness is used to both show the above and illustrate how the method is able to measure the degree of asymmetry. This study applied this approach to two sets of skin lesions in the interest of showing how it can be used, The first group is a recurrent binary shape [17] for which no diagnosis has been made. The second is a set of color images of classified as skin lesions [18]. The experiments show a good correlation both with the prognoses made by a group of dermatologists in the first database, and with a statistical percentage of classification obtained by a second group; in addition, matches the measurements reported in the literature [19, 20].

## Methods

The quantitative measurement of the geometric property known as asymmetry is very similar to that classically used for symmetry. Hence, the first step is to define symmetry in the continuous space, which will be used as the initial point to describe asymmetry in this study.

Taking A and B as two sections of a region C, p and q as two points where p belongs to A and q belongs to B. it can be said that both points are symmetric if, under a mirror transformation relative to one point or plane inside C, both points have the same position. If the overall points of both partitions satisfy the same condition, it can be said that A and B are symmetric and C has a symmetry plane [21]. In the continuous space, a region can have zero, one or several symmetric planes, i.e. a circle. Nevertheless, if a point in the partition does not meet the symmetry condition, it can be said that A and B are asymmetrical.

However, in the digital space, the regions are composed of a set of regular polygons called pixels. There are only three regular polygons that can be used to cover the space: square, hexagon or triangle. Due to technical requirements, the most commonly used polygon is square. Because of the nature of the type of polygon used and according to the definition of symmetry, only rectangular regions can be symmetrical in this space.

The approach used in this research to measure asymmetry begins with the knowledge of the shape, as a digital binary region, which will theoretically be asymmetric. By adhering to the latter condition, the process for finding the major axis is avoided, because the region must be convex and many biological entities do not fulfill this requirement. Nevertheless, if the major axis is found and the digital region is rotated, this would generate small modification in its structure.

This section presents the process for calculating asymmetry, comprising, firstly, the application of one of two methods for calculating the centroid of a digital region either Hu moments or the mean position of the pixels [22].

In some cases, it is necessary to find the angle of the major axis, for which the use of Hu moments is recommended; however, the mean pixel position is adequate for this study. The centroid position can be found using Eqs. 4 and 5.

4 $$ \widehat{r}=\frac{1}{A}{\sum}_{\left(r,c\in R\right)}r $$

5 $$ \widehat{c}=\frac{1}{A}{\sum}_{\left(r,c\in R\right)}r $$

where *r* and *c* are the mean row and column in region *R* respectively, *A* represents the area of the shape.

It should be noted that digital binary images follow the rules of digital topology, which state that a set of discrete elements can create a digital region [23].

**Fig. 1:**
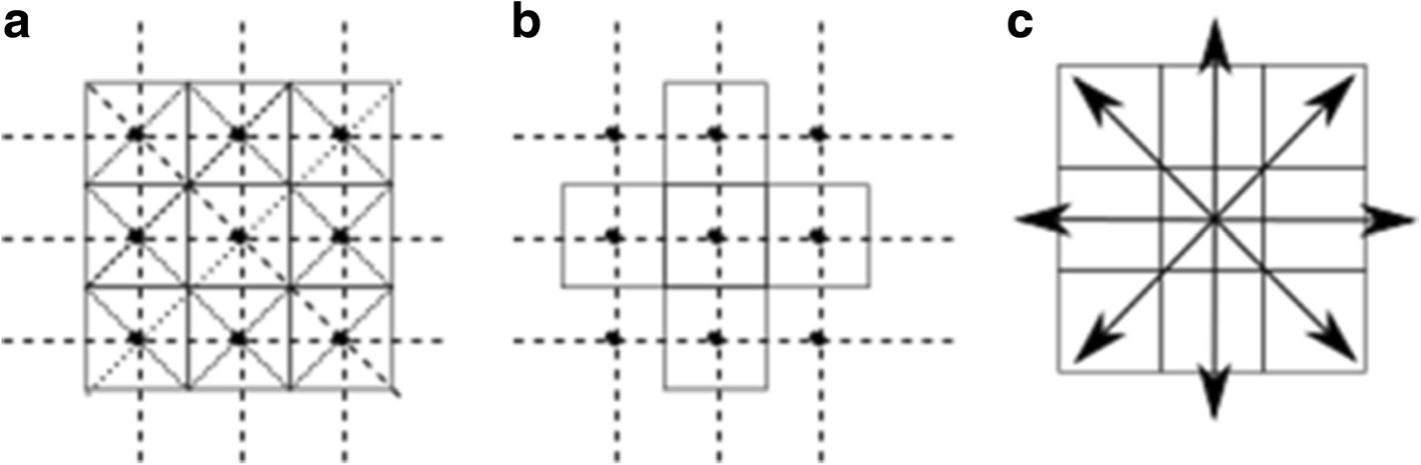
Three types of connectivity in digital space. **a** Eight connectivity and its triangular grill. **b** Four connectivity and its respective square grill. **c** The four possible axes from one position when is used the eight connectivity

**Fig. 2:**
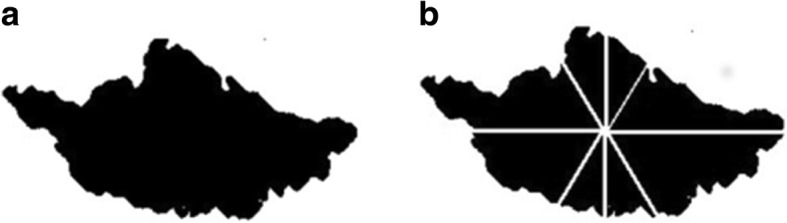
Skin lesion binary representation. **a**. Digital region of a skin lesion with higher possibility to be melanoma (Lee’s database) (**b**). Section of the (**a**) when it is processed by our approach

The element used by the proposed topology is known as connectivity, and is the rule that determines the existence of a region. In the digital space, there are two kinds of connectivity, known as eight and four connectivity. The method proposed in this study uses eight connectivity, which states that, from one position, there are only eight possible directions. Thus, for the objectives of this study, only four axes are possible. Figure 1 shows both connectivity cases, specifically the axes set used for measuring the asymmetry. These four axes are used in the process of segmenting the digital region, in which eight segments or sub-regions are generated. Figure 2 shows the result of this process when it is applied to the digital binary region of a malignant skin lesion. Each sub-region is evaluated in order to produce a compactness value using the Normalised E-Factor (NEF) of Santiago-Montero et al. [24].

Identifying the most compact shape in the digital space, NEF is a shape descriptor that uses the information contained in the border of the digital region, applying a perimeter ratio with a square and the same area. This descriptor is robust to scale, rotation and translation transformation [24].

Table 1 describes the set of values obtained by the NEF of each sub-region of Fig. 2.

**Table 1:** NEF values for each sub-region of Fig. 2 (b)

Region	R1	R2	R3	R4	R5	R6	R7	R8
NEF Value	1.333	1.420	1.313	1.449	1.341	1.470	1.270	1.945

6 $$ NEF=\frac{P_{shape}}{4\sqrt{n}} $$

where *P* is the digital region perimeter and *n* is its area.

Once the compactness values are obtained, these are applied to Eq. 7 with the objective of producing the asymmetry value (Asymmetry_NEF).

The asymmetry value is 0.2499 for the region of Fig. 2.

7 $$ Asymmetry\_ NEF={\sum}_{i=1}^4{\left({NEF}_{Ri}-{NEF}_{Ri+4}\right)}^2 $$

where *NEF*_*Ri*_ is NEF value in region *i*.

**Fig. 3:**
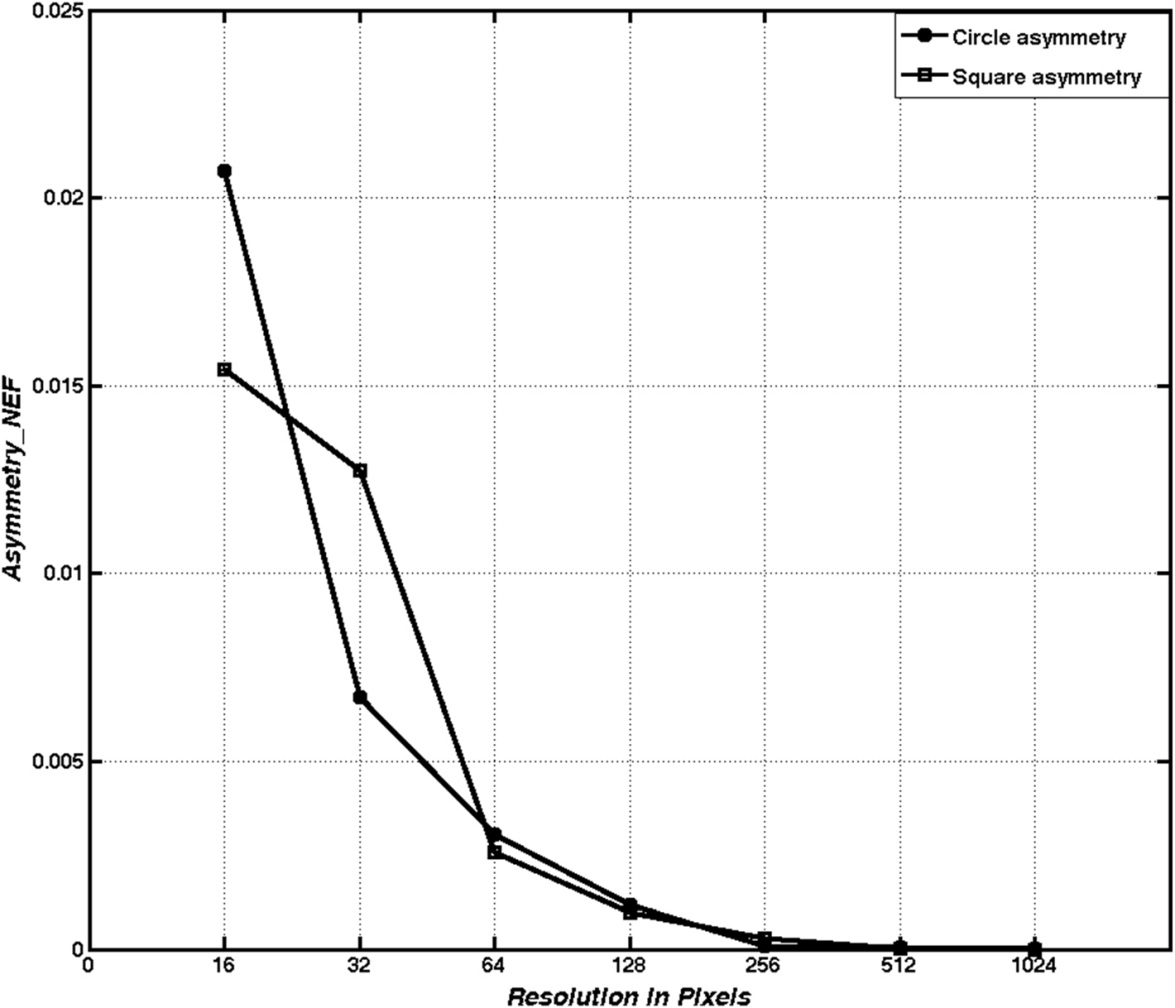
Square and circle Asymmetry_NEF values. Behavior of the measurement when it is applied to a square and circle regions with resolution changes

**Fig. 4:**
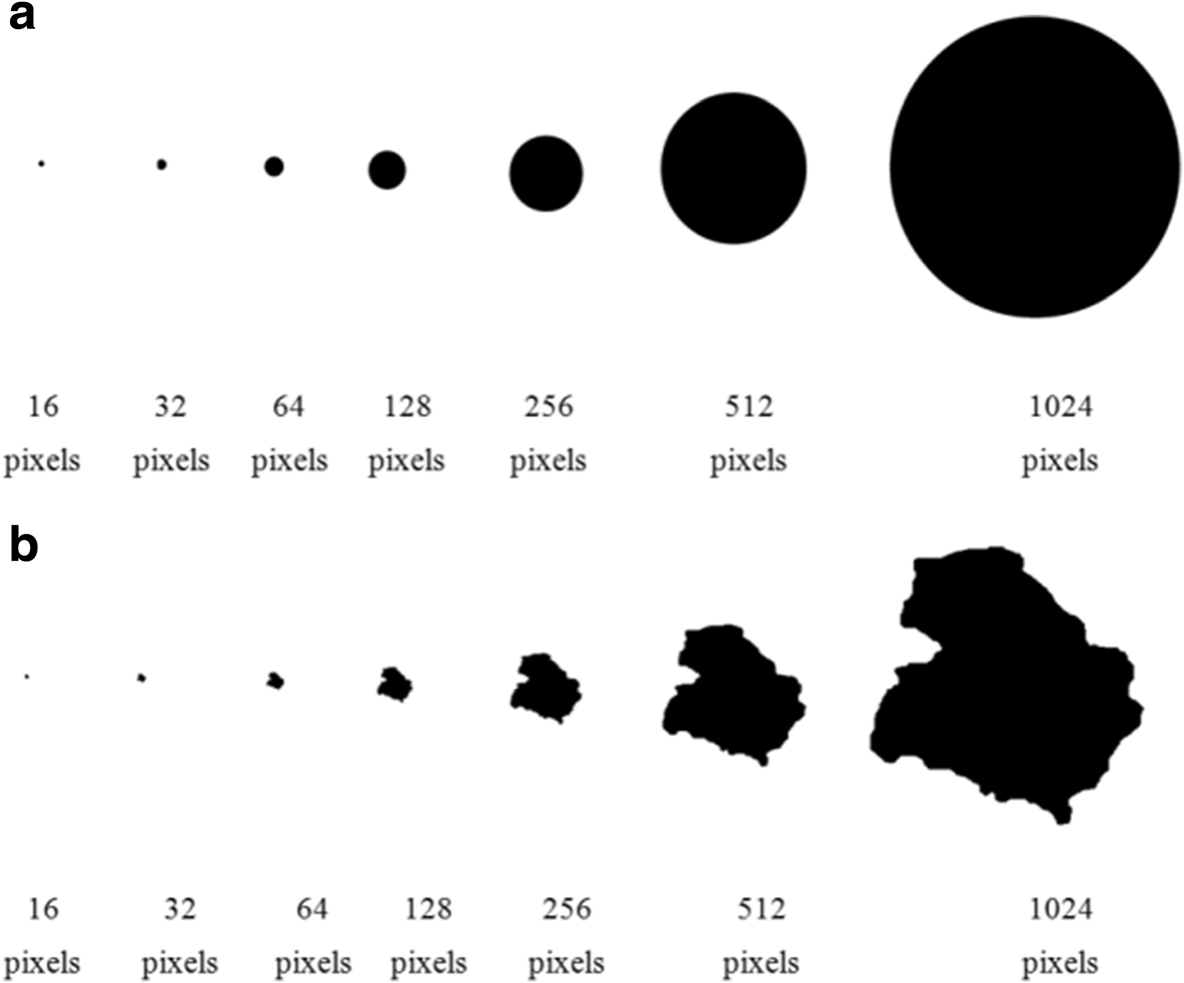
Circle and skin lesion asymmetry values. Circle (**a**) and melanoma (**b**) with different resolution changes

Figure 3 shows that the asymmetric value decreases asymptotically as the resolution increases in geometric forms, such as squares and circles. Fig. 4a shows that these digital forms have a low degree of asymmetry.

Asymmetry measurements show their robustness to resolution variations when tested with an irregular melanoma shape (Fig. 4b). This Figure shows that, in the case of digital regions with a low pixel number, the perimeter contribution made by each pixel is significant.

**Fig. 5:**
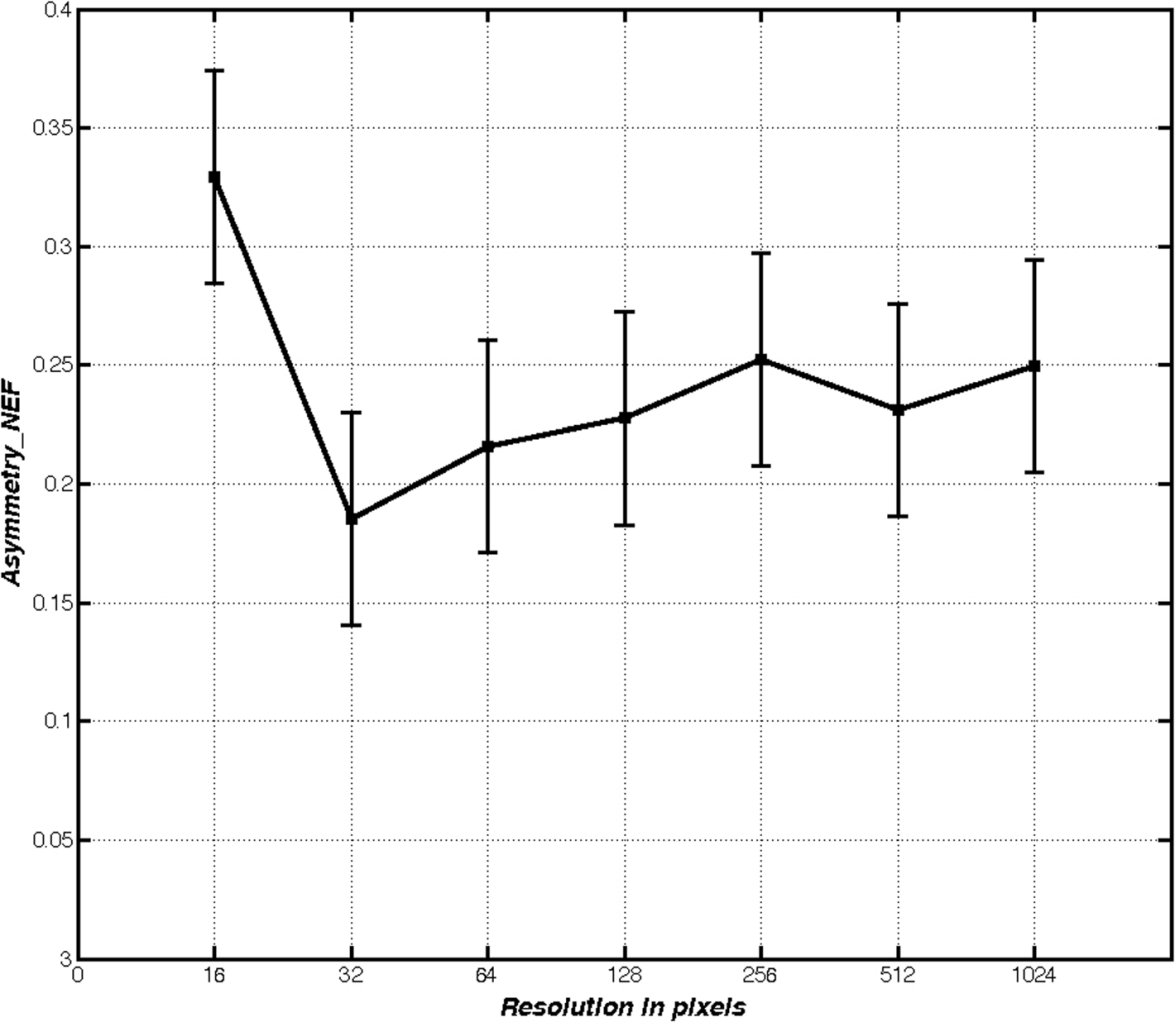
Skin lesion Asymmetry_NEF values bahavior. Asymmetry measurement behavior when it is applied to a digital region of a melanoma shape with scale changes

Figure 5 shows the behavior of the asymmetry values obtained from a malignant skin lesion at different resolutions.

**Fig. 6:**
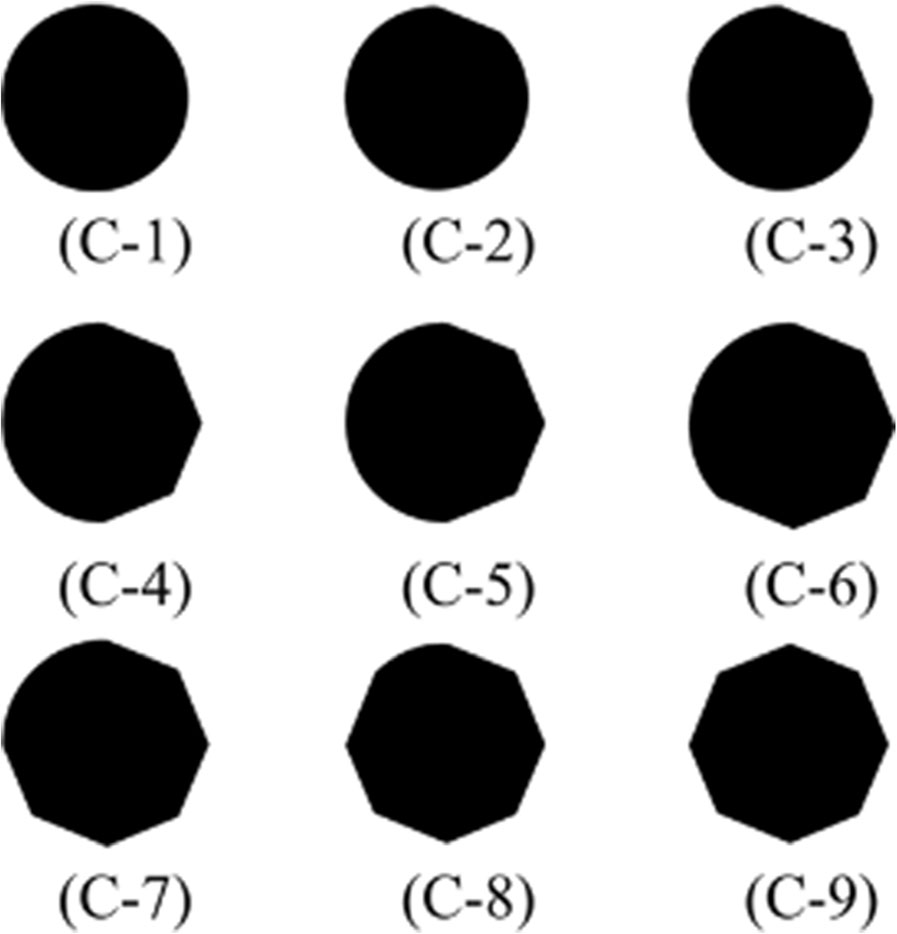
Transformation from circle to octagon. Set of digitized circle without some sections with a border-to-border distance of 512 pixels into a frame of 563 by 545 pixels

**Fig. 7:**
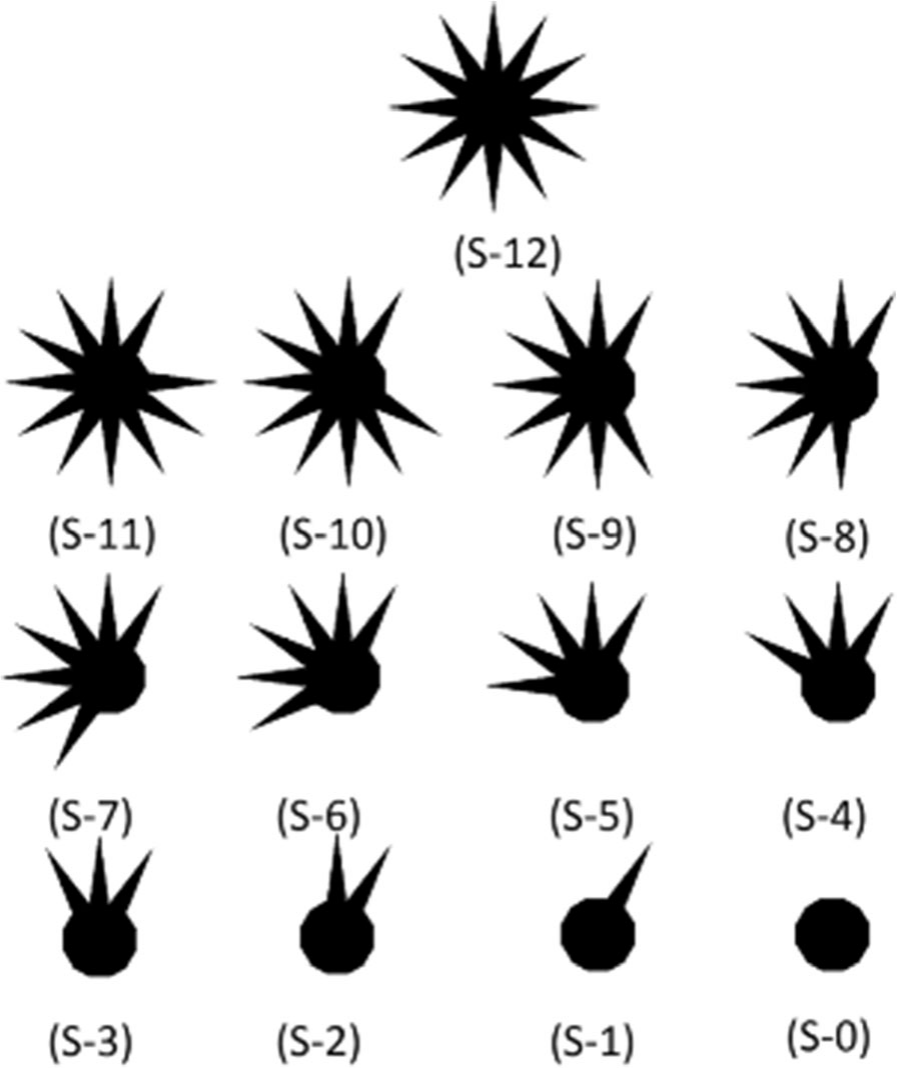
Transformation from 12 peak shape to dodecagon. Set of digital region, which they are removing sections to become in a asymmetric shape to after, again become in a shape with low asymmetry. The digital region was created in a frame of 331 by 331 pixels and 300 pixels of peak-to-peak distance

The second experiment applied on the images in Fig. 6 shows how asymmetry increases and then decreases, a result which provides evidence of a correlation between measurements and the concept of asymmetry. Table 2 shows the test set for the second experiment with its asymmetric values. The experiment was repeated, but with the set of regions that have an irregular border, as shown in Fig. 7. Table 3 shows the set of regions and the asymmetry values obtained by the measurement used in this research, the results of which, once again, show a correspondence with asymmetry shape perception.

**Table 2:** Asymmetry_NEF values of Fig. 6

Shape	C-1	C-2	C-3	C-4	C-5	C-6	C-7	C-8	C-9
Asymmetry_NEF Value	1.333	1.420	1.313	1.449	1.342	1.470	1.270	1.945	0

**Table 3:** Asymmetry_NEF values of Fig. 7

Shape	S-12	S-11	S-10	S-9	S-8	S-7	S-6	S-5	S-4	S-3	S-2	S-1	S-0
Asymmetry_NEF Value	0.002	0.304	1.544	2.900	3.328	3.545	5.149	3.830	4.944	5.665	2.469	0.615	0.0002

## Results

This section reports the results obtained for the proposed method using two skin lesion image databases.

**Fig. 8:**
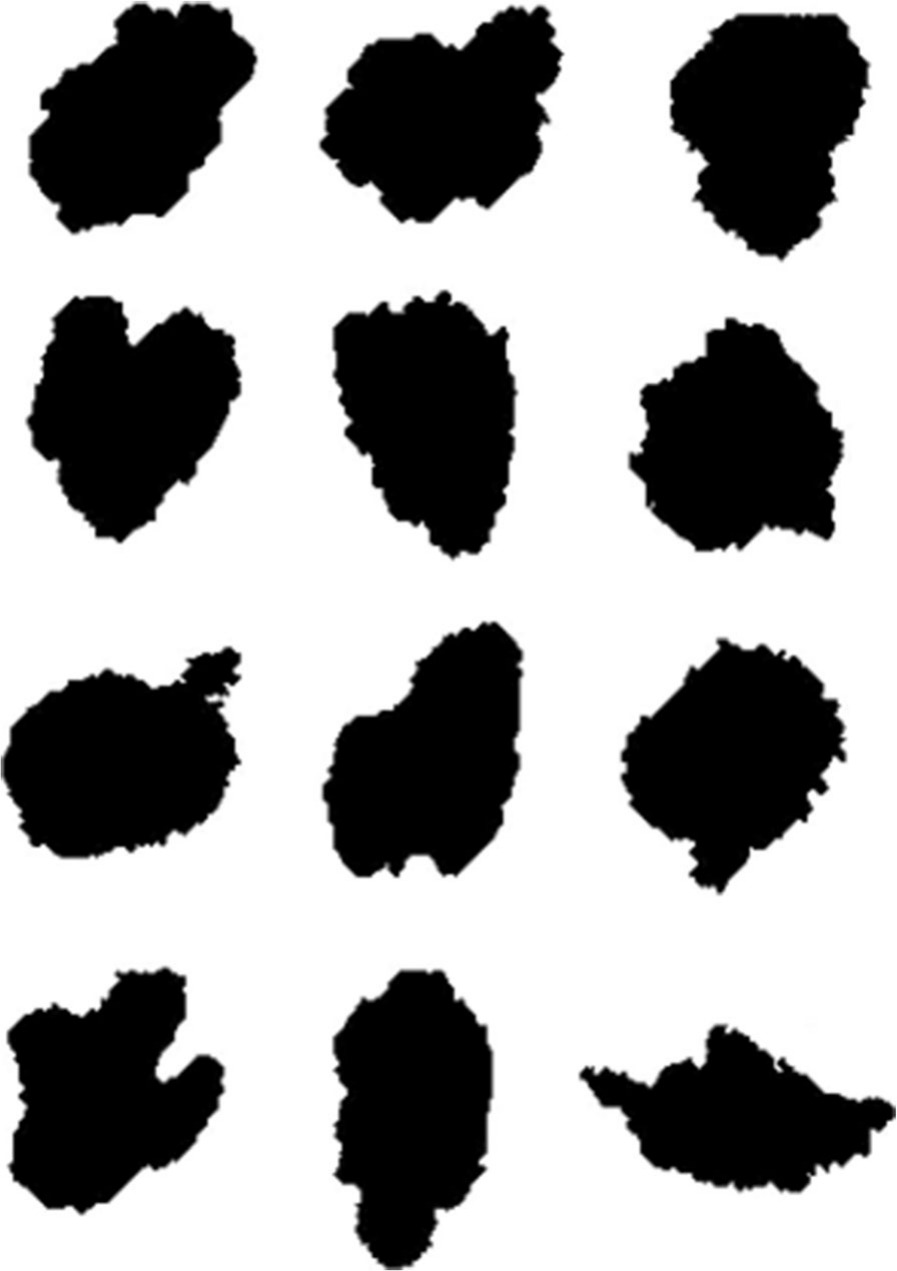
Lesion binary images. Twelve lesion binary images with most Asymmetry_NEF value on Lee’s database

**Fig. 9:**
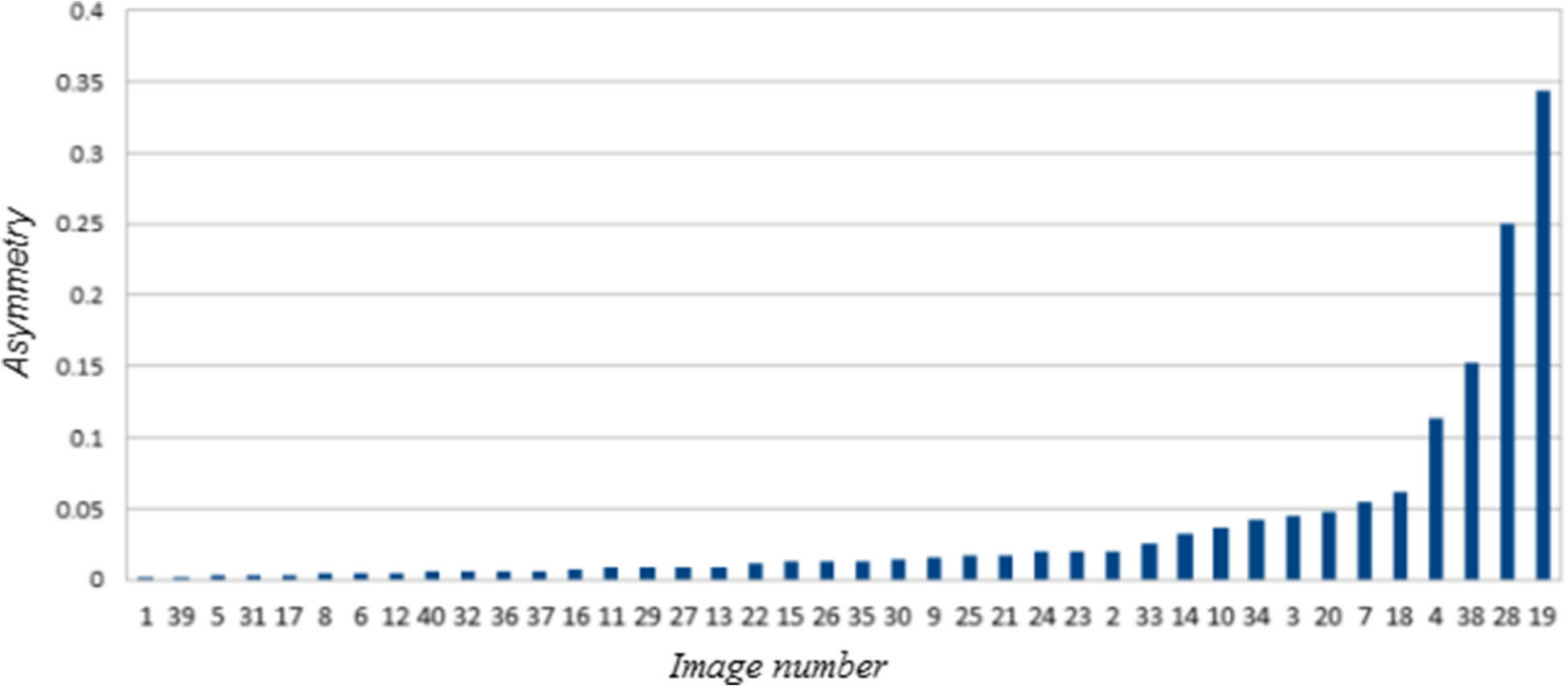
Asymmetry values plot. Binary images sorted according to their Asymmetry_NEF values on Lee’s database

Test 1: A set of 40 digital regions of skin lesions was used as an initial benchmark. This set has become a reference point in the literature on melanoma evaluation because it had been previously evaluated qualitatively by 14 dermatologists using only the appearance of the shape of the lesion [17]. Forty regions were ranked using the proposed asymmetry value; with these ranked values then tested with the average clinical evaluations using the Spearman coefficient obtaining a result of 0.82 (*p* <  0.001). However, the correlation was of 0.98 (*p* <  0.001) for the 12 regions which implies a higher risk of developing melanoma. The set for these regions is shown in Fig. 8, and their asymmetry values are plotted in Fig. 9.

Table 4 shows the Spearman correlation coefficients and the *p*-value of the asymmetry values obtained with the proposed method as compared to the assessments of the 14 dermatologists for both sets of images. In addition, the asymmetric value correlation statistics proposed by Golston et al. [20] (Irrigularity index), Smaoui et al. [19], (Asymmetry index), Cudek et al. [13] (SFA) and Stoecker et al. [9] (Asymmetry_Ref) are presented. Furthermore, with the objective of comparing the predictive potential of the Asymmetry_NEF values with the indices proposed by Golston, Smaoui, Cudek and Stoeker, Table 5 shows, for the five algorithms, the Sensitivity, Specificity and Area under the ROC (Receiver, Operating Characteristic) curve values suggested by Tronstad & Pripp [25] for diagnostic studies. Additionally, Column 4 presents the *p*-values obtained from Fisher’s exact test, in each of which, the sensitivity and specificity values were compared with those values evaluated under the classification criteria of the dermatologists.

**Table 4:** Spearman coefficients of asymmetry measures with methods: Asymmetry_NEF, Irregularity index reported by Golston et al. [20], Asymmetry index proposed by Smaoui & Bessassi [19], Score For Axis (SFA) reported by Cudek et al. [13] and Asymmetry_Ref proposed by Stoecker et al. [9] using the Lee database with 40 images and 12 images considered high risk

Algorithm	Spearman coefficients	*p*-value
Asymmetry_NEF (40 images)	0.82	< 0.001
Asymmetry_NEF (12 images)	0.98	< 0.001
Irregularity index (40 images)	0.80	< 0.001
Irregularity index (12 images)	0.77	0.003
Asymmetry index (40 images)	0.89	< 0.001
Asymmetry index (12 images)	0.70	0.012
SFA (40 images)	0.25	0.114
SFA (12 images)	−0.19	0.553
Asymmetry_Ref (40 images)	0.08	0.611
Asymmetry_Ref (40 images)	−0.01	0.974

**Table 5:** Sensitivity, Specificity and A_ROC_ values measured with the following methods: Asymmetry_NEF, Irregularity index, Asymmetry index, Score For Axis (SFA) and Asymmetry_Ref using the Lee database with 12 images considered as high risk

Algorithm	Sensitivity	Specificity	A_ROC_	*p*-value
Asymmetry_NEF	92%	96.43%	0.94	< 0.001
Irregularity index	33.33%	71.43%	0.54	1.00
Asymmetry index	16.67%	64.29%	0.40	0.2448
SFA	55%	78.57%	0.45	0.1298
Asymmetry_Ref	55%	78.57%	0.45	0.1298

Test 2: This test used the PH^2^ database reported by Mendonca et al. [18]. Which consists of 200 images in an 8-bit RGB format and an average resolution of 768 × 560 pixels and 20× magnification. There is a binary representation, handmade, for each image in this set. Eighty images were classified by experts as common nevus, eighty as atypical nevus and forty as melanoma. Each image has an asymmetry measurement in one of three classes: 0 representing fully symmetric; 1 representing symmetric on one of its axis; 2 representing fully asymmetric. As there were 117, 31 and 52 samples, respectively; the cases with greater asymmetry values turned out to be the most malignant lesions belonging to Class 2. This classification was used to compare the asymmetry values of the class comprising the images classified by experts as 0 and 1, with the asymmetry values conformed by the images classified as 2.

**Fig. 10:**
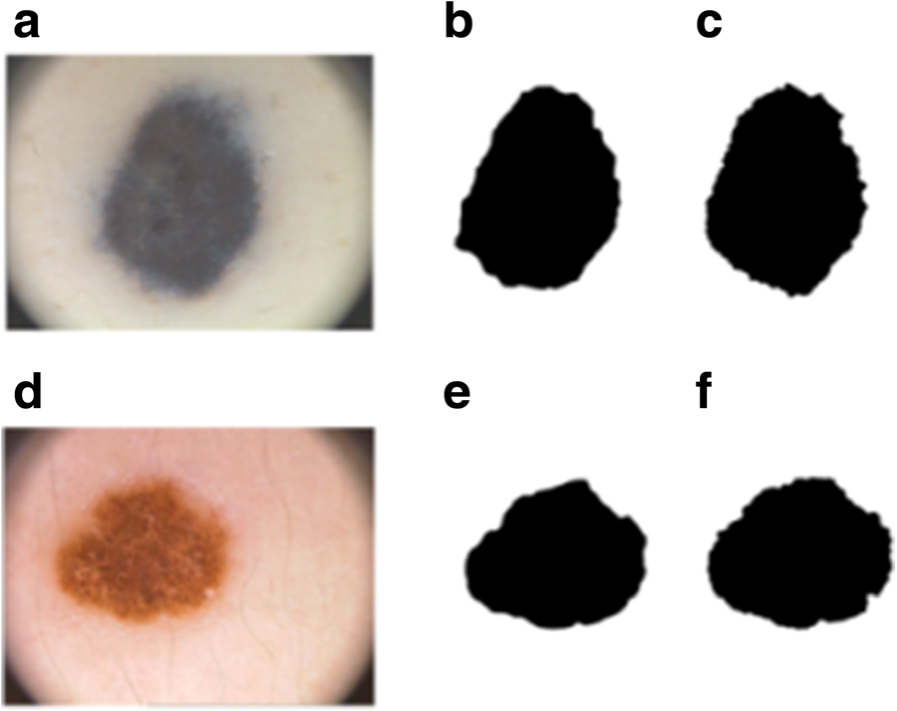
Skin cancer images. Skin picture (**a**) (**d**), manual (**b**) (**e**) and automatic thresholding (**c**) (**f**) methods on skin images

The purpose of this test was to evaluate our proposal considering two threshold method techniques. In the first, the manual method, 14 dermatologists segmented the skin lesion area by hand to obtain the asymmetry borders. In the second, the automatic method, the segmentation method was used to obtain the segmented images and automatically evaluate the asymmetry values [26]. A visual practical comparison between both methods is shown in Fig. 10, which shows that the mean Asymmetry_NEF values are greater than Class 2 with both the manual and automatic threshold methods.

Table 6 shows the mean values for Class 0–1 and Class 2 with manual and automatic threshold methods. It is important to note the significant difference between the average values for both methods of classification.

**Table 6:** Mean and variance values for Class 0–1 and Class 2 with manual and automatic thresholding methods

Class/Thresholding method	Asymmetry_NEF mean values
Class 0–1/Manual	0.043
Class 2/Manual	0.097
Class 0–1/Automatic	0.341
Class 2/Automatic	2.209

Table 7(rows 2 and 3) shows the Wilcoxon rank-sum test values for Class 0–1 and 2, which is labeled Class 2, taking into account the method proposed, as well as those proposed by Golston et al. [20], Smaoui et al. [19], (Asymmetry index), Cudek et al. [13] (SFA) and Stoecker et al. [9] (Asymmetry_Ref). It is important to note that the most significant *p*-value corresponds to the average Asymmetry NEF values, which enables a better classification of the Class 0–1 images compared to Class 2.

**Table 7:** Wilcoxon rank sum test for equal medians values for Class 0–1 and Class 2 with manual and automatic thresholding methods. *H*_0_ : *μClass*_0 − 1_ = *μClass*_2_

Algorithm/Thresholding method	*H*_*0*_	*p*-value	Cohen’s d
Asymmetry_NEF/Manual	0 (Rejected)	0.01	−0.408
Asymmetry_NEF/Automatic	0 (Rejected)	< 0.001	−0.828
Irregularity index/Manual	0 (Rejected)	0.8772	−0.486
Irregularity index/Automatic	0 (Rejected)	0.2157	−0.414
Asymmetry index/Manual	1 (Accepted)	< 0.001	−1.084
Asymmetry index/Automatic	1 (Accepted)	0.0325	−0.24
SFA/Manual	1 (Accepted)	0.0086	−0.45
SFA/Automatic	1 (Accepted)	0.0247	−0.435
Asymmetry_Ref/Manual	0 (Rejected)	0.9678	−0.051
Asymmetry_Ref/Automatic	0 (Rejected)	0.6388	−0.128

This criterion was applied because the most malignant lesion belongs to Class 2. The main goal of this experiment is to show the potential of the approach proposed. To summarize, two thresholding methods were used, a manual method taken from the PH^2^ database and the automatic method described in [26].

Alternatively, the statistical values for comparing the means of both data groups are shown in Table 7 (Rows 4–7), generated by using the Irregularity Index and Asymmetry Index methods.

A similar analysis of the predictive potential of the index proposed, undertaken with the Lee database, is shown in Table 8, taking into account the PH^2^ database. Moreover, it is possible to note that the Asymmetry_NEF has a better relationship with the dermatologists’ classification criteria than the indices proposed by Golston, Smaoui, Cudek and Stoecker.

**Table 8:** Sensitivity, Specificity and A_ROC_ values measured with the following methods: Asymmetry_NEF, Irregularity index, Asymmetry index, Score For Axis (SFA), and Asymmetry_Ref using the PH^2^ database

Algorithm	Sensitivity	Specificity	A_ROC_	*p*-value
Asymmetry_NEF	59.62%	85.81%	0.72	< 0.001
Irregularity index	32.69%	76.35%	0.54	0.2042
Asymmetry index	32.69%	76.35%	0.54	0.2042
SFA	30.77%	75.68%	0.53	0.3641
Asymmetry_Ref	32.69%	73.35%	0.54	0.2042

It can be seen that, for the databases used in this study, the p-value obtained via Fisher’s exact test indicates that the specificity of the diagnosis achieved using the Asymmetry_NEF value does not significantly differ from the diagnosis made by the dermatologists that participated in the classification of melanoma in each of the images from the two databases.

## Discussion

In order to show the utility of this measurement technique, two sets of digital images of skin lesions were tested.

The Spearman coefficient obtained once the 40 asymmetry values were compared with the average clinical evaluation was slightly less than the Spearman coefficient reported by Lee et al. [17], who compared the Overall Irregularity Index (OII) with the average clinical evaluation obtaining 0.82 and 0.88, respectively. In the case of the Most Significant Irregularity Index (MSII), Lee et al. reported a.

Spearman coefficient of 0.81. However, the Spearman coefficient obtained in this paper, once the 12 regions with a higher risk of developing melanoma were analyzed, was 0.98; which implies that the asymmetry value proposed here strongly correlates with the evaluation undertaken by experienced dermatologists. In addition, the proposed asymmetry value had higher correlation values when compared with the Irregularity index for cases comprising 40 and 12 images. However, when compared to the Asymmetry index, it presented a correlation coefficient value when both indices were compared with the 40 images while presenting a significantly lower value, when it was compared to the 12 images with the highest asymmetry value (see Table 4).

With regard to the PH^2^ database, the descriptive statistics (Table 5) and the Wilcoxon median test (Table 6) both showed that the Asymmetry_NEF values correlated well with both human perception in evaluating the asymmetry of skin lesions and the asymmetry values automatically obtained using the method proposed by Abuzaghleh et al. [26]. Cohen’s *d* values presented a significant difference between the medians of the two classes using two threshold methods, thus demonstrating the potential of the approach proposed in this study. Regarding to the Irregularity Index, the average scores obtained were very similar to both the Manual and Automatic methods. However, in the case of the Asymmetry Index, it was not possible to determine significant differences between the two groups in terms of both methods (Table 6).

Esteva et al. [27] used a CNN as a machine-based learning for the classification of skin lesions based on the texture rather than the shape of the images. Moreover, as this approach cannot be naturally implemented in the actual CAD systems, the classification system needs to be changed completely. As a result, there has been a tendency to implement this CAD system on mobile devices, which due to the high computational cost of the CNN has, it seems been difficult. However, the proposed method is more likely to able to be implemented on mobile applications due to the reduced computational cost. Consequently, the proposed asymmetry measure could be an input variable for the learning machine reported by Esteva et al. [27], making a more efficient and effective system for skin lesion classification.

It is the author’s opinion that the proposed method can be extended to other shape descriptors in the space or frequency domain, which would be able to increase their efficiency and effectiveness. Research is underway in those areas, and will be reported in subsequent papers.

## Conclusions

The measurement proposed in this study is dominated by the rules of digital topology and symmetry definition. The method proposed demonstrates the adequate correlation between its quantitative values and the concept of asymmetry. Moreover, it has a suitable tolerance to scale transformation in the digital space. This study demonstrates that, with a lack of digital space and applied under eight connectivity, the digital shapes with an irregular border can be symmetrical.

Hence, in accordance with the symmetry definition of digital space, the square and rectangular shapes present the lowest grade of asymmetry. Therefore, it can be concluded that the proposed method is a simple but effective way to parameterize the concept of asymmetry.

Experiments with geometric shapes (squares and circles) and melanoma images with higher asymmetry values showed that, for melanoma images, the Asymmetry_NEF value did not vary significantly in terms of resolution.

Finally, the sensitivity, specificity, ROC area and *p*-value measurements in Tables 5 and 8 show a significant relationship between the dermatologists’ classification criteria and the NEF asymmetry values. The advantages of the method proposed by this study are highlighted in comparison with the methods by Golston et al. [20], Smaoui & Bessassi [19], Score For Axis (SFA) reported by Cudek et al. [13] and Asymmetry_Ref proposed by Stoecker et al. [9]. This proposes the possibility for using the Asymmetry_NEF as a reliable asymmetry measure for the classification of various biological tissues.

## Abbreviations

CADS: Computer Aided Diagnosis System
NEF: Normalised E-Factor
TDV: Total Dermatoscopic Value
